# A Cross-species Model of Dual-Task Walking in Young and Older Humans and Rats

**DOI:** 10.3389/fnagi.2020.00276

**Published:** 2020-09-02

**Authors:** Abbi R. Hernandez, Steven P. Winesett, Quinten P. Federico, Sonora A. Williams, Sara N. Burke, David J. Clark

**Affiliations:** ^1^Department of Neuroscience, College of Medicine, University of Florida, Gainesville, FL, United States; ^2^Department of Applied Physiology and Kinesiology, College of Health and Human Performance, University of Florida, Gainesville, FL, United States; ^3^College of Medicine, University of Florida, Gainesville, FL, United States; ^4^Department of Aging and Geriatric Research, College of Medicine, University of Florida, Gainesville, FL, United States; ^5^Brain Rehabilitation Research Center, Malcom Randall VA Medical Center, Gainesville, FL, United States

**Keywords:** aging, cognition, executive function, walking, brain, prefrontal cortex, dual-task

## Abstract

**Introduction**: Dual-task walking is common in daily life but becomes more difficult with aging. Little is known about the neurobiological mechanisms affecting competing cognitive demands. Translational studies with human and animal models are needed to address this gap. This pilot study investigated the feasibility of implementing a novel cross-species dual-task model in humans and rats and aimed to establish preliminary evidence that the model induces a dual-task cost.

**Methods**: Young and older humans and rats performed an object discrimination task (OD), a baseline task of typical walking (baseline), an alternation turning task on a Figure 8 walking course (Alt), and a dual-task combining object discrimination with the alternation task (AltOD). Primary behavioral assessments including walking speed and correct selections for object discrimination and turning direction. In humans, left prefrontal cortex activity was measured with functional near-infrared spectroscopy (fNIRS).

**Results**: Human subjects generally performed well on all tasks, but the older adults exhibited a trend for a slowing of walking speed immediately before the turning decision for Alt and AltOD compared to baseline. Older adults also had heightened prefrontal activity relative to young adults for the Alt and AltOD tasks. Older rodents required more training than young rodents to learn the alternation task. When tested on AltOD with and without a 15-s delay between trials, older rodents exhibited a substantial performance deficit for the delayed version on the initial day of testing. Old rats, however, did not show a significant slowing in walking speed with increasing task demand, as was evident in the young rats.

**Discussion**: This study demonstrates the feasibility and challenges associated with implementing a cross-species dual-task model. While there was preliminary evidence of dual-task cost in both humans and rats, the magnitude of effects was small and not consistent across species. This is likely due to the relative ease of each task in humans and the walking component in rats not being sufficiently challenging. Future versions of this test should make the cognitive tasks more challenging and the motor task in rats more complex.

## Introduction

Control of walking in humans requires a significant contribution from cognitive resources, particularly for complex walking conditions. In daily activities, walking is often performed simultaneously with cognitive tasks, which is known as dual-task walking. Dual-tasking generally yields a performance decrement (dual-task cost) for one or both tasks as compared to single-task performance (Smith et al., 2017). In older adults, dual-task walking can lead to dyscoordination and slowing of movement that increases the risk of accidental slips, trips, and falls (Beauchet et al., 2009; Asai et al., 2020). Enhancing or maintaining a dual-task walking function is therefore an important goal for promoting safe walking in older adults.

A barrier to improving dual-task walking function is that relatively little is known regarding the basic neurobiological mechanisms of managing competing-cognitive demands. Mechanisms that could potentially be investigated with animal models but that are not feasible in humans include recording of single neurons and neuronal networks, directly altering neurotransmitter systems, and lesioning or chemogentic manipulation of specific brain regions (Watanabe and Funahashi, 2018). It may be possible to develop dual-task paradigms that can be used across humans and animals, such that findings can be translated as well as reverse translated to identify, refine, and test mechanistic hypotheses, as well as develop new interventions. Interestingly, many of the common behavioral assays utilized in rodent studies to investigate age-related cognitive decline require locomotor tasks of walking (Olton and Samuelson, 1976; Barnes, 1979; Hernandez et al., 2015, 2018a; Johnson et al., 2017), or swimming (e.g., water maze studies; Morris, 1981; Gallagher et al., 1993). While rodent behavioral assays have been instrumental in identifying the hippocampus (Rosenzweig and Barnes, 2003) and prefrontal cortex (Burke and Barnes, 2006; Morrison and Baxter, 2012) as brain regions vulnerable in aging, analysis of age differences has primarily focused on cognitive aspects of task performance. Great care is taken to use measures that are not influenced by age-related differences in physical performance. When physical assessments are included, it is evident that aged rats swim slower than their younger counterparts (e.g., Gallagher et al., 1993; McQuail and Nicolle, 2015; Hernandez et al., 2020b). Similarly, walking speed is reduced in aged rodents relative to young animals (Shen et al., 1997; Burke et al., 2014; Cowen et al., 2020). Thus, rodent behavioral paradigms naturally combine cognitive and locomotor tasks and are therefore analogous to what is called dual-task walking in the human literature, suggesting that evaluating motor performance in rats in the context of cognitive tasks that vary in difficulty could be a useful model. Moreover, prior evidence indicates that complex walking and cognitive functions rely on overlapping large scale brain networks (Leisman et al., 2016). Notably, the neural networks that both complex walking and higher cognition likely involve the prefrontal cortex and hippocampus, which are also among the most susceptible structures to age-related dysfunction (Morrison and Baxter, 2012), indicating that dual-task walking across species could have a common mechanism.

The objective of this pilot study was to investigate the feasibility of implementing a novel cross-species dual-task paradigm and to establish preliminary evidence that the paradigm induces a dual-task cost in both rats and humans. The current experiments combine a continuous alternation walking task with an object recognition task, and was initially designed for rodents to assess age-related decrements of dual-task performance. The human version of the dual-task was then developed to be roughly analogous to the rodent behavioral task. It is important to acknowledge upfront some of the challenges of designing a cross-species paradigm. Unlike humans, who can be instructed to follow a particular procedure, rodents must be incrementally trained over days to weeks to follow the study procedures. Therefore, humans experience a novel exposure to some of the task demands while rodents have already become accustomed to the task. Another notable difference is that humans likely dedicate greater attentional resources to walking due to the inherently unstable nature of bipedal gait and potentially serious consequences of task failure, including injurious falls. In contrast, a rodent walks close to the ground with quadrupedal support and is at low risk of an injurious fall. Nevertheless, there is potentially great scientific value to validating a cross-species dual-task paradigm that can be used in translational lines of research that seek to discover treatments for age-related decrements in dual-task walking. The primary hypotheses of this study were that both species would exhibit dual-task costs affecting walking and that these costs would be more pronounced in the older subjects.

## Methods

### Experiment 1: Human Subjects

#### Human Participants

Seventeen elderly and eight young participants enrolled in this study. Demographic information and functional performance data are presented in Table 1. Assessments were conducted at a research laboratory in a university setting, and all participants signed an informed consent form that was approved by the local Institutional Review Board (study number IRB201300835).

**Table 1:** Human subject demographics and functional status.

	Older adults	Young adults
Age (years)	66.7 + 5.4	22.9 + 2.8
BMI (kg/m^2^)	27.3 + 5.0	22.8 + 4.7
10MWS (meters/s)	1.28 + 0.2	1.28 + 0.1
ABC Scale (%)	95.3 + 5.2	97.1 + 3.1
Sex (female/male)	8/4	7/1
BBS (points out of 56)	54.9 + 1.5	
SPPB (points out of 12)	11.8 + 0.4	
3MS (points out of 100)	94.2 + 8.9	

*BMI, body mass index; 10MWS, 10-meter walk speed; ABC, Activities Specific Balance Confidence; BBS, Berg Balance Scale; SPPB, Short Physical Performance Battery; 3MS, Modified Mini-Mental State Exam*.

#### Object Memorization

**Figure 1:**
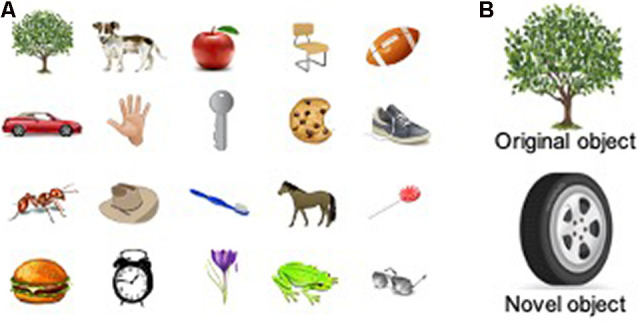
Object memorization and object discrimination for humans. **(A)** An example group of images used during object memorization, in which human participants were given 45 s to view and memorize a group of 20 objects. **(B)** An example pair of objects used during the object discrimination assessment, in which human participants were shown a familiar object (from the original group of 20 images) and a novel object that the participant had never seen.

While seated in a chair, participants were given 45 s to view and memorize a group of 20 objects (illustrations). An example set of illustrations is shown in Figure 1A.

#### Object Discrimination (OD)

Participants were shown a pair of object illustrations during an encoding phase. During the OD testing phase, one object was from the original list of twenty (Figure 1B, top), and the other was a novel object that the participant had never seen (Figure 1B, bottom). Participants were instructed to discriminate between the two objects by pointing to whichever object they recognized from the prior list. If a selection was not made within 5 s, it was scored as an incorrect response. The page was then turned to show the next pair of objects, and this process was repeated for 10 pairs.

#### Standard Walking (Baseline)

**Figure 2:**
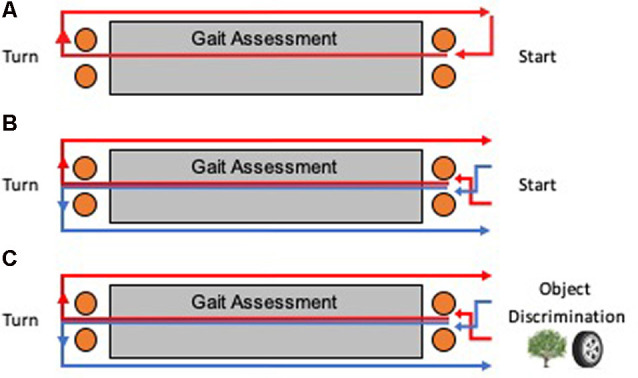
Testing apparatus for humans. Multiple laps around a course were taken for each task, with an instrumented mat recording spatial and temporal gait measurements during the first straight portion of each lap. A bird’s eye view of the course is shown for each task. **(A)** Baseline walking involved only right turns for each lap. **(B)** Continuous alternation walking (Alt) involved a Figure 8 shaped pattern of alternating turns. **(C)** Continuous alternation walking with object discrimination (AltOD) combined the Alt task with the object discrimination task. Object discrimination was performed at the start of each new lap.

Participants walked at their preferred self-selected speed for three consecutive 15-m laps on a rectangular course (Figure 2A). Participants were instructed to take a right turn at the halfway point of each lap. On the first straight portion of the course, an instrumented mat captured gait spatial and temporal measurements. Orange cones were placed one meter before and after the mat to ensure that participants turned at a consistent location and to ensure that participants walked in a straight line over the length of the mat.

#### Continuous Alternation Walking (Alt)

Participants walked at their preferred self-selected speed for 10 consecutive 15-m laps on a rectangular course (Figure 2B). Participants were instructed to follow a Figure 8 walking pattern, such that the halfway point of each lap required alternating left and right turns (a complete Figure 8 required two laps). As a control procedure for similarity to the dual-task procedure (next section), the participant paused for 5 s at the end of each lap to look at a blank binder page. As with the *Baseline* walking task, an instrumented mat captured gait spatial and temporal measurements along the first straight portion of each lap and orange cones were used to mark turning locations.

#### Continuous Alternation Task Walking with Object Discrimination (AltOD)

Participants followed the same procedure as described above for “Continuous Alternation Walking” (Alt) and “Object Discrimination” (OD), but with the two tasks interwoven (AltOD; Figure 2C). For the object discrimination component, the participant stopped walking after each lap and was shown the pair of illustrations.

#### Performance Assessments

For both the continuous alternation task and object discrimination, responses were categorized as “correct,” “incorrect,” or “indecision.” Incorrect responses were determined objectively when a participant turned the wrong direction during walking (e.g., two left turns in a row on the Figure 8 course) or pointed to the wrong illustration during object discrimination. Decisions taking longer than 5 s on the object discrimination task were also considered incorrect. Indecision on the walking tasks was defined subjectively by a pause or major slowing before making a correct turn. Indecision on the object discrimination task was defined as a response taking longer than 3 s, or switching responses before making a correct selection. Following each task, participants were asked to report a perceived task difficulty on a scale of 1–10, where 1 is very easy and 10 is extremely difficult.

To further assess walking performance differences between groups and across tasks, spatiotemporal gait data were measured by an instrumented mat (GAITRite, CIR Systems, Havertown, PA, USA) located on the first straight path of each lap (the center portion of the Figure 8). The temporal resolution for individual footfall data is 1 ms. The primary outcomes of interest were walking speed, as well as the extent to which walking slowed before turning at the halfway point of each lap (where the left vs. right-turning decision occurred). To assess the latter, we measured changes in gait parameters for the final two steps on the instrumented mat relative to the earlier steps on the mat during that same lap. The gait parameters of interest were step time and step length (which together determine step velocity).

#### Prefrontal Activity Measured by Functional Near-Infrared Spectroscopy

For all tasks, a functional near-infrared spectroscopy device (fNIRS; Portalite, Artinis Medical Systems Inc.) was used to measure the recruitment of the left frontopolar aspect of the prefrontal cortex during all tasks. The left prefrontal cortex is important to multitasking performance (Burgess et al., 2007). fNIRS estimates neuronal activity in the underlying cortex by calculating hemodynamic changes due to neurovascular coupling (Perrey, 2014). The optodes were secured to the forehead by double-sided adhesive. To further minimize movement artifact in the signal and reduce ambient light, optodes and wires were secured by a fabric headband. Equipment cables were also secured to the upper back to minimize artifacts from cable movement. A diode emitted infrared light at continuous wavelengths of 760 nm and 810 nm. The source-detector separation distance was 3 cm. Changes in prefrontal oxygenated hemoglobin concentration (O_2_Hb) were estimated with the modified Beer-Lambert Law. Data were sampled at 10 Hz and saved directly to a memory card in the data acquisition unit, and later downloaded to a computer for analysis. Before beginning each task, participants rested quietly in a standing position for approximately 1 min to provide a baseline level of prefrontal activity. Participants were not told exactly when the walking task would begin, to prevent an anticipatory increase in prefrontal recruitment. Resting baseline prefrontal activity was quantified as the average hemoglobin concentration during the final 10 s of the rest period that preceded each task. For the active period, prefrontal activity was averaged over a 30-s period that began 7 s after task onset to allow for cerebral blood flow changes to stabilize (Vitorio et al., 2017).

Prefrontal O_2_Hb data were analyzed with custom programs created with Matlab version R2015a (Mathworks, Natick, MA, USA). O_2_Hb signals were smoothed with a 0.14 Hz low pass filter to remove the potential effects of heart rate and respiration. A wavelet filter was then applied to remove non-physiological deflections of the signal such as movement artifacts (Cooper et al., 2012). The fNIRS outcome measure was changed in oxygenated hemoglobin concentration (ΔO_2_Hb) between the resting baseline period and the active period within each task, calculated using the following equation: *Prefrontal ΔO_2_Hb = Active O_2_Hb − Resting O_2_Hb*.

#### Statistics

All data are expressed as group means ± standard error of the mean (SEM) unless otherwise reported. Analyses of task performance, self-reported task difficulty, gait parameters, and fNIRS were conducted with separate repeated-measures ANOVAs (RM-ANOVA) with the between-subjects factors of age (young or older groups), repeated measurements of the task, and interaction effect. Mauchly’s test of sphericity was used to test the assumption that variances of the differences between all combinations of the conditions were equal. The Greenhouse–Geisser correction was applied if this assumption was violated. *Post hoc* analyses were conducted with standard or paired *t*-tests, as appropriate. All analyses were performed with JMP Pro 14.0.0 (SAS Institute Inc.).

### Experiment 2: Rodent Analogue

#### Subjects and Handling

Young (beginning at 4–7 months) and aged (beginning at 23–24 months) male (*n* = 10) and female (*n* = 10) Fisher 344 × Brown Norway F1 (FBN) Hybrid rats from the NIA colony at Charles River were used in this study (*n* = 5/group; 20 rats total). Each rat was housed individually and maintained on a 12 h light/dark cycle and all behavioral testing was performed in the dark phase. To encourage appetitive behavior in object discrimination experiments, rats were calorically restricted, receiving 10–30 g (1.9 kcal/g) moist chow daily based on rats’ relative body conditions on a scale of 1–5, with 3 being ideal, 1 being emaciated, and 5 being morbidly obese (Hickman and Swan, 2010). Water was provided *ad libitum*. Rats’ body weights were recorded daily, and body condition was assessed and recorded weekly throughout restricted feeding. The body condition score was assigned based on the presence of palpable fat deposits over the lumbar vertebrae and pelvic bones (Hickman and Swan, 2010). Animals with a score under 2.5 were given additional food to promote weight gain. Shaping began once rats reached approximately 85% of their baseline weights or had a body condition score of three. All procedures were following the NIH Guide for the Care and Use of Laboratory Animals and approved by the local Institutional Animal Care and Use Committee (#201708644).

#### Apparatus and Habituation

**Figure 3:**
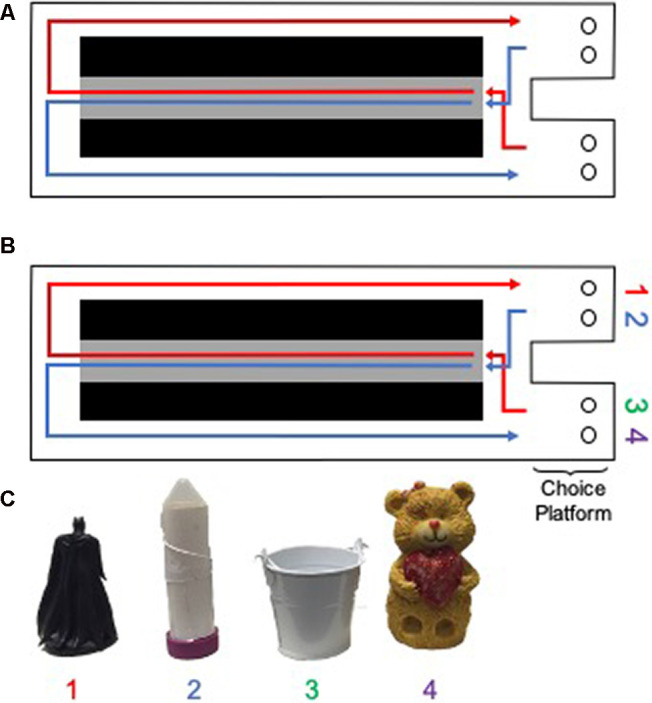
Testing apparatus and objects for rats. A Figure 8 shaped maze was used for **(A)** the continuous alternation and **(B)** object discrimination-based testing in rats (birds-eye view of the maze is shown). Gray area indicates the center arm, from which walking speed data were obtained. Black areas indicate negative space. Circles within the choice platform indicate food wells positioned below possible object locations. **(C)** Objects used in the rat version of the alternation object discriminations (AltOD). Note objects 1 and 2 were always utilized within the right choice platform, whereas objects 3 and 4 were always utilized within the left object choice platform. Rats had to displace the object by pushing it to retrieve a possible food reward located in the well beneath the object.

All behavioral testing occurred on a Figure 8-shaped maze (see Figure 3) 67.5 inches long and 25 inches wide. The maze was constructed from wood and sealed with waterproof white paint. The center arm was made of clear acrylic. The choice platforms each contained two food wells (2.5 cm in diameter) that were recessed into the maze floor by 1 cm. All arms were 4 inches wide. The choice platforms were contained within 7.5 cm raised walls and the right arm was contained within 20 cm high raised halls, but the center and left arms did not have walls. Thus, the arms of the maze had an asymmetry such that only the right arm was enclosed while the animals were relatively more exposed to the middle and left arms. To dampen the influence of extraneous noise on behavior, a white noise machine was used during behavioral training and testing. Rats were habituated to the testing apparatus for 10 min a day for two consecutive days, with Froot Loop pieces (Kellogg Company, Battle Creek, MI, USA) scattered throughout the maze to encourage exploration.

#### Alternation and Object Discrimination Tasks

Following habituation to the testing apparatus, rats were trained to alternate between the left and right arms of the maze (Figure 3A). Correct alternations were rewarded with ~1/2 of a Froot Loop placed randomly in either well within the chosen platform. Because learning effects may be masked by procedural acquisition or differences in the amount of time it took aged rats to become appetitively motivated, the first 2 days of alternation training were excluded from the analysis. Over these 2 days, several aged rats completed minimal laps as food restriction was being implemented. When rats were alternating correctly ≥80% of the time on two consecutive days, they began training on the object discrimination task, beginning with an alternation object discrimination dual-task (AltOD) for three consecutive days. In addition to correctly alternating between the left and right arms of the maze, rats had to choose between two objects located above the food well within each choice platform (Figures 3B,C). Distinct object pairs were presented in each arm and one object from each pair was consistently rewarded. Objects were placed over the medial and lateral food wells pseudorandomly across trials and a different order was used each day, so there was no detectable pattern to the object locations. All object discrimination-based training consisted of 20 trials per day each day, testing on consecutive days. Incorrect alternations resulted in the removal of objects (no object choice presented to the rat) until the next turn on which they correctly alternated throughout the maze. If a rat alternated incorrectly three times in a row, they were guided to the correct side on the subsequent turn to ensure objects were encountered on an adequate number of trials per training session. On the first day of training with objects during AltOD, the objects only partially covered the food wells on the first eight trials (four per arm) to facilitate the rats learning that food was hidden beneath objects. This was analogous to the encoding phase of the OD testing in human study participants.

Alternations alone and in conjunction with the AltOD task were repeated for three consecutive days each. Following these 6 days of testing, an additional 3 days of testing was conducted in which a 15-s delay was imposed between trials to further tax working memory on the alternation component of the task. Because the correct choice on the OD component of the task was not dependent on the previous trial, in the absence of a dual-task cost, the delay should not affect an animal’s ability to select the correct object. During this AltOD task with a delay, following object choice (or entrance into a choice platform for the alternation task), a barricade was placed outside the object-choice area for 15 s to prevent rats from entering the center arm of the maze until the delay period had passed.

Videos of all behavioral testing were recorded for offline analysis by experimenters blinded to animal age. The ambulatory velocity while the rat traversed the center arm of the Figure 8 maze during each task was calculated using the recordings by determining the time it took rats to traverse the median 110 cm of the center arm of the maze. A primary outcome of interest was to assess walking speed and the degree to which it was affected by the addition of higher cognitive load. Ambulatory velocity while traversing the center arm of the maze was calculated while rats were alternating throughout the maze, regardless of direction, to assess baseline velocity with no cognitive load. Additionally, velocity was assessed on the final day of testing on AltOD and Delayed AltOD.

#### Statistical Analysis

All data are expressed as group means ± standard error of the mean (SEM) unless otherwise reported. The percent of correct object choices or alternations for each task were analyzed using a two-factor ANOVA with the between-subjects factors of age (two levels: young and aged) and sex (two levels: male and female). Tasks in which the percent of correct object choices or correct alternation choices were compared across multiple days were analyzed using repeated measures-ANOVAs (RM-ANOVA) with the between-subjects factors of sex and age. Values outside of the interquartile range were excluded from the analysis. All analyses were performed with Statistical Package for the Social Sciences (SPSS) v25 or GraphPad Prism version 7.03 for Windows (GraphPad Software, La Jolla, CA, USA).

## Results

### Experiment 1: Dual-Task Walking in Humans

#### Participant Characteristics

Compared to the younger group, the older group had significantly older age (*t*_(24)_= 21.2, *p* < 0.001, *d* = 2.01) and the exhibited a trend for higher body mass index (*t*_(24)_= 2.07, *p* = 0.05, *d* = 0.84). However the groups did not differ for preferred 10-m walking speed (*t*_(24)_= 0.08, *p* = 0.93, *d* = 0.04), or the Activities Specific Balance Confidence Scale (*t*_(24)_= 0.86, *p* = 0.40, *d* = −0.38).

#### Task Performance and Perceived Difficulty

**Figure 4:**
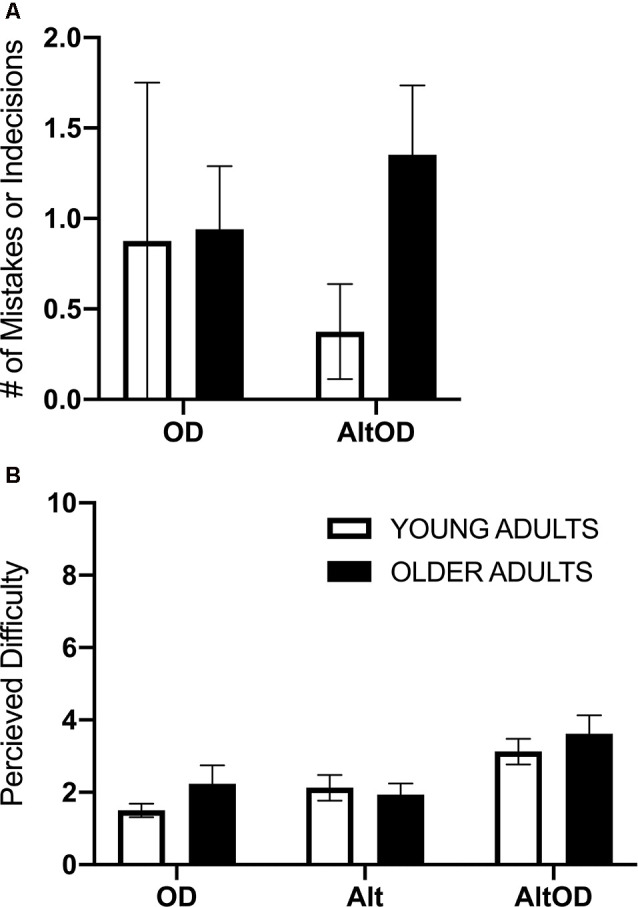
Human task performance and perceived difficulty. **(A)** The summed number of trials with mistakes or indecision for object discrimination is shown for each task. **(B)** Perceived difficulty based on self-reporting rating from 1 to 10 is shown for each task. OD, object discrimination; Alt, alternation walking; AltOD, combined alternation walking and object discrimination.

Participants generally performed well on the walking and cognitive tasks, with relatively few incorrect responses or indecision. Therefore, incorrect responses and indecision were summed within each task and participant. Separate models were run to assess object discrimination performance in OD and AltOD and to assess turning performance in Alt and AltOD. In both cases, the models yielded no statistically significant findings for Group, Task, or interaction (Figure 4A). For self-reported task difficulty there was a main effect of Task (*F*_(2,22)_ = 15.0; *p* < 0.0001, $\eta_{\text{p}}^{2}$ = 0.58), but not for Group (*F*_(1,23)_ = 0.36; *p* < 0.56, $\eta_{\text{p}}^{2}$ = 0.02) or the interaction (*F*_(2,22)_ = 0.82; *p* = 0.45, $\eta_{\text{p}}^{2}$ = 0.07). *Post hoc* analysis revealed that participants found *AltOD* to be significantly more difficult than *OD* (*t*_(24)_= 5.16; *p* < 0.0001, *d* = 1.04) and *Alt* (*t*_(24)_= 4.87; *p* < 0.0001, *d* = 0.98). However, overall the perceived difficulty of the tasks was low in both age groups (Figure 4B).

#### Walking Spatiotemporal Measurements

**Figure 5:**
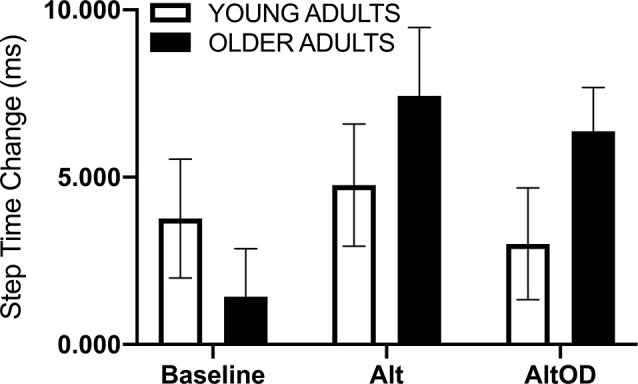
Human gait temporal data. The step time change was calculated as the mean step time from the last two steps before the turn minus the mean step time from all earlier steps in the same lap. Alt, alternation walking; AltOD, combined alternation walking and object discrimination.

For walking speed there was no significant effect of Group (*F*_(1,20)_ = 1.37; *p* = 0.25, $\eta_{\text{p}}^{2}$ = 0.06), Task (*F*_(2,19)_ = 0.63; *p* = 0.54, $\eta_{\text{p}}^{2}$ = 0.06), or interaction (*F*_(2,19)_ = 0.02; *p* = 0.98, $\eta_{\text{p}}^{2}$ = 0.002). For step time just prior to making a turn, a positive value indicates that participants took longer duration steps immediately prior to making the turn as compared to earlier in that same lap (Figure 5). The effect of Group (*F*_(1,20)_ = 1.01; *p* = 0.33, $\eta_{\text{p}}^{2}$ = 0.05) and Task (*F*_(2,19)_ = 1.46; *p* = 0.26, $\eta_{\text{p}}^{2}$ = 0.13) were not significant, but there was a trend for a Group × Task effect (*F*_(2,19)_ = 3.07; *p* = 0.07, $\eta_{\text{p}}^{2}$ = 0.24) with older adults appearing to show more pronounced slowing as indicated by lengthening of step time prior to making the turn as the tasks became more complex (i.e., *AltOD* greater than *Baseline* or *Alt*). A similar analysis was conducted for step length but no statistically significant effects were observed.

#### Functional Near-Infrared Spectroscopy

**Figure 6:**
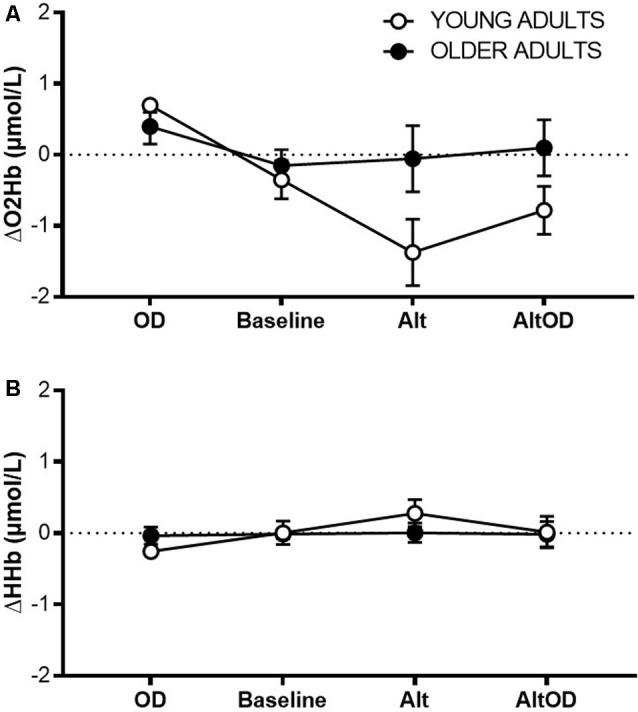
Prefrontal cortical activity measured by fNIRS. **(A)** Task-related change in oxygenated hemoglobin concentration (O_2_Hb; **A**) and deoxygenated hemoglobin concentration **(B)** from left Brodmann Area 10 for each task. OD, object discrimination; Alt, alternation walking; AltOD, combined alternation walking and object discrimination.

For prefrontal ΔO_2_Hb (Figure 6A) there was a violation of sphericity (Mauchly criterion = 0.46, *p* = 0.03), so an adjusted univariate Greenhouse–Geisser correction was applied to the model. There was a significant effect of Task (F_(2.1,34.9)_ = 6.68; *p* = 0.003, $\eta_{\text{p}}^{2}$ = 0.28) and Group × Task interaction (F_(2.1,34.9)_ = 4.37; *p* = 0.02, $\eta_{\text{p}}^{2}$ = 0.20). ΔHHb (Figure 6B) did not differ by Task (F_(3,16)_ = 1.83; *p* = 0.18, $\eta_{\text{p}}^{2}$ = 0.28) or Group × Task interaction (F_(3,16)_ = 1.10; *p* = 0.38, $\eta_{\text{p}}^{2}$ = 0.19). Within the young group, prefrontal ΔO_2_Hb was significantly greater for *OD* than for *Baseline* (*t*_(5)_= 3.09; *p* = 0.027, *d* = 0.79), *Alt* (*t*_(5)_= 3.99; *p* = 0.011, *d* = 1.61), and *AltOD* (*t*_(5)_= 3.74; *p* = 0.014, *d* = 1.53). In contrast, the older adults showed no statistically significant difference in prefrontal ΔO_2_Hb between any of the tasks. One possible exception was a trend for greater ΔO_2_Hb for *OD* compared to *Baseline* (*t*_(12)_= 1.98; *p* = 0.07, *d* = 0.53).

### Experiment 2: Dual-Task Walking in Rodents

#### Impaired Learning of Alternation Behavior in Aged Rats

**Figure 7:**
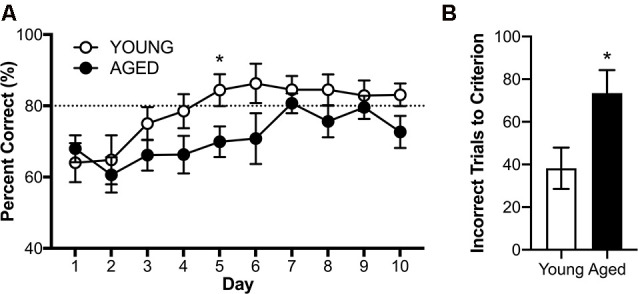
Alternation acquisition in the Figure 8 maze. **(A)** Daily performance across days 3–10 of training demonstrate aged rats are impaired at the acquisition of the alternation rule. **(B)** When comparing the number of incorrect trials before reaching criterion young rats required significantly fewer trials than aged rats. All values are group means ± SEM. The dotted line represents criterion performance. *p < 0.05.

RM-ANOVA with the dependent variable of percent correct turns on days 3–10 of testing revealed a significant main effect of day (*F*_(7,112)_ = 2.41; *p* = 0.02, $\eta_{\text{p}}^{2}$ = 0.71), but no interactions between day and age or sex (Figure 7A, $\eta_{\text{p}}^{2}$ < 0.46 for all comparisons). While there was no main effect of sex (*F*_(1,16)_ = 2.87; *p* = 0.11, $\eta_{\text{p}}^{2}$ = 0.15), there was a significant main effect of age (*F*_(1,16)_ = 5.65; *p* = 0.03, $\eta_{\text{p}}^{2}$ = 0.26), such that young rats acquired the alternation rule more quickly than the aged animals. Because there were no significant effects of sex on performance, male and female performance was considered together for each age group for further analysis of alternation behavior.

The first day on which any group met the threshold for criterion performance (≥80% correct on two consecutive days) was day 5 for the young group (mean % correct = 84.40 ± 4.50). On this day, young rats overall performed significantly better than aged rats (*t*_(18)_ = 2.33; *p* = 0.03, *d* = 1.04). On the tenth day of testing, there was no longer a significant effect of age (*t*_(18)_ = 1.90; *p* = 0.07, *d* = 0.87). An alternate way to investigate the effect of age on alternation acquisition is to investigate how many incorrect trials each rat underwent before reaching criterion performance (≥80% correct on two consecutive days). Young rats made significantly fewer errors before reaching criterion performance than aged rats (*t*_(18)_ = 2.44; *p* = 0.03, *d* = 1.10; Figure 7B), further demonstrating an age-related impairment in acquisition of the working memory component of the task.

#### Object Discrimination During Dual-Tasking Is Impaired More in Aged Rats Than Young Rats

**Figure 8:**
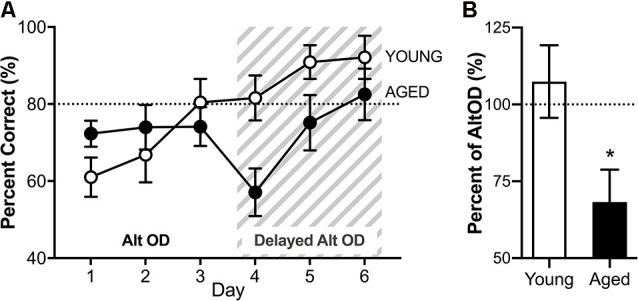
Object discrimination task performance. **(A)** There were no differences across age groups on the alternation object discrimination (AltOD) task performance (days 1-3). When the difficulty is increased further by adding a delay between trials (gray striped area, days 4–6), there is again no significant difference with age across the 3 days of testing. **(B)** However, when the difference in performance on the last day of no delay AltOD is compared to the first day of delayed AltOD, aged rats had a significant decline imposed by the delay while the young rats did not. All data are group means ± SEM. The dotted line indicates performance on an easier version of the task represented in the graph. *p < 0.05.

RM-ANOVA for all 3 days of AltOD testing revealed no significant main effects of day (*F*_(2,30)_ = 2.45; *p* = 0.10, $\eta_{\text{p}}^{2}$ = 0.29) and no interactions between day and age or sex (Figure 8A). Furthermore, performance did not differ across age (*F*_(1,15)_ = 0.488; *p* = 0.50, $\eta_{\text{p}}^{2}$ = 0.3) or sex (*F*_(1,15)_ = 1.68; *p* = 0.21, $\eta_{\text{p}}^{2}$ = 0.10), nor did these factors interact (*F*_(1,16)_ = 0.07; *p* = 0.79). Because there were no significant effects of sex on performance, male and female performance was considered together for each age group for further analysis of AltOD behavior. This is consistent with previously published data that young and aged rats perform similarly on simple object discrimination tasks when the objects do not share features (Hernandez et al., 2015; Johnson et al., 2017; Maurer et al., 2017).

Immediately following the 3rd day of AltOD, the same alternation object discrimination task was employed with the addition of a 15-s delay imposed between alternation trials. Notably, this delay increases the working load for alternating in which the turning direction from the previous trial determines the correct choice in the subsequent trial. It should, however, not impact OD performance in which the correct object choice was incrementally learned over the preceding 3 days and is independent of one trial to the next. When the delay was imposed, all rats continued to perform near ceiling levels on the continuous alternation component of the task. The delay, however, did appear to affect the OD performance. RM-ANOVA for all 3 days of testing revealed a significant main effect of day on selection of the correct object (*F*_(2,26)_ = 10.57; *p* < 0.001, $\eta_{\text{p}}^{2}$ = 0.52), and a trend towards an age impairment with old rats making more incorrect object selections (*F*_(1,13)_ = 3.79; *p* = 0.07, $\eta_{\text{p}}^{2}$ = 0.23). Age and day did not significantly interact (*F*_(1,26)_ = 0.68; *p* = 0.52, $\eta_{\text{p}}^{2}$ = 0.06). Because the two tasks were implemented back to back, performance for selecting the correct object on the last day of AltOD (day 3) was compared to performance on the following day during which the delay was first imposed (day 4). Performance with delay is expressed as the percent of trials that the correct object was chosen on the final day of AltOD (Figure 8B). Aged rats were significantly more impaired by the imposition of a delay than young rats (*t*_(18)_ = 2.48; *p* = 0.02, *d* = 0.95). In fact, young rats were not significantly impaired by the delay (*t*_(9)_ = 0.633; *p* = 0.54, *d* = −0.05) but imposing a delay did significantly decrease performance in aged rats (*t*_(9)_ = 3.03; *p* = 0.014, *d* = 1.07). This indicates that while the delay did not affect young or aged rats’ abilities to correctly alternate across trials, it did interact with aged rats’ abilities to retrieve the correct object choice during the dual-task with a delay. This suggests that there may be a cognitive dual-task cost such that performance on the OD component of the task was compromised in aged rats to maintain performance on the continuous alternation task.

#### Aged Rats Walked Slower Than Young Rats but Did Not Exhibit a Dual-Task Cost in Motor Performance

**Figure 9:**
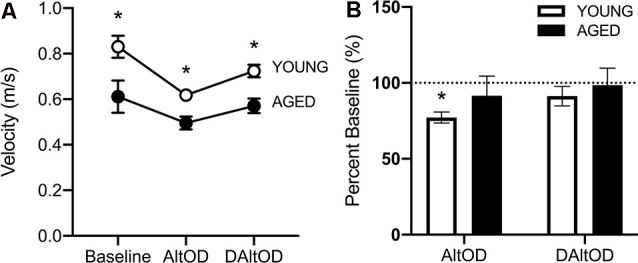
Ambulatory velocity within the center arm of the maze during behavioral tasks. **(A)** Aged rats were significantly slower during all behavioral testing than young rats. **(B)** Young rats were significantly slower during AltOD than at baseline, but aged rats did not differ. Neither group was significantly slower during delayed AltOD (DAltOD). The dotted line indicates baseline performance. All data are group means ± SEM. *p < 0.05.

For the final day of testing on AltOD and Delayed AltOD, RM-ANOVA on these days with the between-subjects factors of age and sex revealed no significant effect of sex (*F*_(1,11)_ = 3.97; *p* = 0.07, $\eta_{\text{p}}^{2}$ = 0.43), therefore all rats were averaged together per age group. The aged rats were significantly slower than young (*F*_(1,13)_ = 19.62; *p* = 0.001, $\eta_{\text{p}}^{2}$ = 0.71; Figure 9A), and there was a significant effect of task type on velocity (*F*_(3,39)_ = 13.38; *p* < 0.001, $\eta_{\text{p}}^{2}$ = 0.47), indicating that the addition of object discrimination decreased ambulatory speed down the center maze of the arm. However, there was not a significant interaction between task and age group on walking speed (*F*_(2,26)_ = 0.14, *p* = 0.87, $\eta_{\text{p}}^{2}$ = 0.03). The lack of an interaction effect suggests that the motor cost of dual-tasking was not more pronounced in aged rats, which is inconsistent with observations from humans. To further examine the potential dual-task cost within age groups, the walking velocities during the two dual tasks (AltOD and delayed AltOD) were calculated as a percent of walking velocity during the Alt only condition in which the cognitive load was minimal. Young rats had significantly reduced velocity during AltOD (*t*_(9)_ = 4.51; *p* < 0.003, *d* = 1.60) but not Delayed AltOD (*t*_(7)_ = 1.63; *p* = 0.14; *d* = 1.92). In contrast, aged rats did not have a significant reduction in walking speed between Alt only and Alt with either OD task (*t*_(8)_ > 1.52; *p* > 0.17, *d* < 0.52 for both comparisons; Figure 9B). This suggests that young, but not aged, rats exhibited a decline in motor performance associated with the dual-task cost.

## Discussion

The results of this study show that a cross-species dual-task walking paradigm is feasible, with preliminary results suggesting that both humans and rats could show a dual-task cost. The pattern of dual-task costs in the different age groups, however, were not consistent between humans and rats. The primary hypotheses were partially supported by evidence of dual-task costs affecting walking, particularly in older adults. Not all measures showed dual-task cost, and the effects that were observed were generally small. This is likely due to the high functional status of the older groups (humans and rodents) and the relative ease of the cognitive tasks for humans and the walking task not being sufficiently complex for rats. Another consideration for comparing data from humans and animal models of dual-task behavior is the necessary use of food restriction in animal models to encourage appetitive behavior. Feeding patterns strongly influence health outcomes across species (Mattson et al., 2014). Human study participants likely have a range of dietary preferences and eating patterns that are difficult to control for and likely to confound data, particularly in older adults that are more likely to exhibit metabolic dysfunction. In animal models, energy consumption patterns can be tightly controlled and optimized to not adversely impact health. The once-daily feeding regime used in the current rat experiments has been shown to improve metabolic function compared to *ad libitum* feeding, which promotes excessive energy consumption and obesity in rats (Hernandez et al., 2020a). Thus, food restriction in animals may offer additional experimental control, facilitating investigations into the neurobiological mechanisms of dual-task costs across the lifespan. Finally, the statistical analyses were generally underpowered given the pilot nature of the study and small sample sizes. The results nevertheless provide valuable data that can contribute to the experimental and statistical design of future larger studies. Below we discuss these points in more depth, along with other lessons learned.

### Selection of Motor and Cognitive Features for Dual-Tasking

Walking is the most widely studied task in the context of cognitive-motor dual-tasking in humans, and is crucial to physical independence in the aging population. Walking also lends itself well to studying dual-tasking in both humans and rats because it can be performed relatively easily for extended periods. Additional tasks can be readily layered on top of walking, and there are well-established performance metrics (e.g., walking speed) and measurement approaches for quantifying dual-task costs. One difficulty in using walking metrics as a primary performance measure across species, however, is the difference between bipedal and quadrupedal gait and the risk of an injurious fall being a unique feature of bipedal gait.

Two cognitive tasks were selected for the study. A criterion for selection of a cognitive task was that it should be reasonability applicable to “real world” functioning in humans. One of the selected tasks was a working memory task in which the subjects should follow a Figure 8 path during walking, alternating left and right turns at the end of a central walkway. Working memory is a component of executive function (Bizon et al., 2012; Beas et al., 2013), which is a cognitive domain that is closely related to walking function in older adult humans (Yogev-Seligmann et al., 2008). The continuous alternation task is similar to remembering a set of walking directions through a building corridor or grocery store, such that left and right turns are required to reach a particular destination. The second task was an object discrimination task in which the subjects identified one object from a pair, based on retrieval of information from recognition memory. This task is similar to remembering a shopping list and choosing the correct item when there are multiple varieties to choose from. There are precedent for utilizing object discrimination tasks to assess cognitive aging in both humans (Ryan et al., 2012; Stark et al., 2013, 2015) and rodents (Burke et al., 2010, 2012; Johnson et al., 2017).

### Findings From Human Participants

The participants enrolled in this study were healthy and high functioning. The older group exhibited good performance on assessments of functional walking, balance, and cognition. Self-reported perceived difficulty of the *AltOD* task was significantly greater than for *Alt* or *OD* alone, though in all cases the difficulty ratings were quite low. This fact, combined with the high functional status of the older adult group, likely contributes to the small effects observed for objective measures of dual-task cost across tasks and between groups. The most notable finding was a marginally significant Group × Task effect when assessing the slowing of gait parameters at the “decision point” of the Figure 8 walking course, just before participants had to take the left or right turn (Figure 5). This may be a promising measure to include in future studies. The direction of effect suggests that older adults tend to slow more during the more complex walking tasks, and more so than young adults.

Also notable was the Task × Group interaction effect for changes in prefrontal ΔO_2_Hb during walking, as measured by fNIRS. Only for young adults, higher average ΔO_2_Hb was observed during the *OD* task compared to all of the walking tasks, including *AltOD* where the object discrimination periods were distributed between periods of walking. Since neural control of walking is considered to be relatively “automatic” in healthy young adults (Clark, 2015; Hawkins et al., 2018), attentional demands and prefrontal activity are expected to be low. Even though dual-tasking during *AltOD* would likely have invoked some use of prefrontal resources (Holtzer et al., 2011, 2015), the majority of the task period (e.g., about 75%) was spent walking. So the average recruitment across the task period was dominated by the low level of prefrontal recruitment during walking. In older adults, the findings were markedly different. Prefrontal ΔO_2_Hb was maintained at a similar level across all of the tasks, which suggests that older adults dedicated comparable levels of attention during walking as during the object discrimination task. This yielded a relatively heightened ΔO_2_Hb in older vs. young during Alt and AltOD tasks (Figure 6A), suggesting compensatory recruitment in older adults that may have aided task performance as indicated by only small performance decrements relative to young adults (Reuter-Lorenz and Cappell, 2008; Clark et al., 2014). A methodological weakness of this approach is that only a small region of cortical activity was recorded. Further, fNIRS data were not corrected for possible task-related changes in systemic blood flow (such as through the use of short-separation fNIRS channels or physiological monitoring of heart rate and blood pressure). Limitations in technology for recording brain activity during walking in humans is a major motivating factor for pursuing animal models that can reveal neurobiological mechanisms of dual-task walking performance.

### Findings From Rodents

Aged rats exhibited poorer performance when dual-tasking, but only when a delay was imposed. Moreover, the aged rats’ walking speed was not significantly slowed by adding cognitive tasks. Interestingly, the young rats did show evidence of the motor cost of dual-tasking. Because aged rats walk more slowly than young animals at baseline, they were not further slowed by the addition of the AltOD tasks. These data are critical for the development of a translational model of dual-task assays involving a motor component and suggest that a physical assessment other than simple walking may need to be implemented to examine the neurobiology of motor/cognitive dual tasking in rats. While there have been rat and non-human primate (Gray et al., 2017) based dual tasks (for review see Watanabe and Funahashi, 2018), none of these tasks was a motor/cognitive dual-task like the tasks described here.

Our previous work has shown there are age-related differences in neuronal activity within the medial prefrontal cortex (mPFC), the rodent homolog to the human dorsolateral prefrontal cortex. A previous study reported that while sitting in the home cage not engaging in a task, aged animals exhibit greater baseline activity within the mPFC relative to young animals as well as greater activation during both alternation and object discrimination tasks even when performance was comparable across age groups (Hernandez et al., 2018b). The reduced dynamic range of prefrontal cortical activity in aged compared to young animals is consistent with the fNIRS data from humans in the current experiments. Together these data suggest that behavioral performance on the dual-task presented here results from an impaired ability to recruit prefrontal cortical circuits in aged subjects relative to young. This impaired recruitment of neural activity could result from higher baseline activity that limits the dynamic range of task-related PFC activity to meet task demands (Reuter-Lorenz and Cappell, 2008) that may emerge from a competition for necessary executive resources to support dual tasking.

In rats, the largest differences in walking speed during a cognitive task relative to baseline walking were observed within young subjects. This could be because aged rats exhibited slower walking speeds at baseline and thus were already exhibiting symptoms of compensating for enhanced load relative to young rats before increasing cognitive load. Another possibility of the lack of a significant decrease in ambulatory speed in aged rats with increasing task demand is the aforementioned limited risk of falling in quadrupedal animals during simple walking.

### Determining the Validity of a Cross-species Model and Recommendations for Future Dual-Task Protocols

The development of novel cross-species behavioral paradigms requires the demonstration of face validity, predictive validity, and target validity within the animal model when compared to observations from humans (Denayer et al., 2014). In the context of examining the neurobiology of dual-tasking across the lifespan, face validity would be the detection of a decrement in motor task performance (i.e., walking) that emerges when a second cognitive task is performed simultaneously. Furthermore, for such a task to provide utility to understanding mechanisms of physical and cognitive decline in aging, this decrement should be more pronounced in older groups, as is observed in human study participants (Beurskens et al., 2014; Brustio et al., 2017a,b). The current experimental design failed to establish face validity. Although older adults in the human component of the current experiments had more pronounced slowing at that decision point on the alternation maze when dual-tasking, a similar finding was not observed in the rats. Only the young rats showed a significant decline in walking speed between the single task and the dual-task condition. There are two potential, not mutually exclusive explanations for this observation. First, the aged rats walked slower overall and thus has a reduced potential to reduce their speeds as cognitive load increased by adding a secondary task. Second, unlike older adults, aged rats have a relatively low risk of injury from a fall. This may result in executive resources not being allocated to simple walking. Thus, future experiments should employ a physical assessment that does pose a modest risk of injury should a fall occur. One possible approach would be to use a horizontal ladder walking task in which foot placement must carefully target the rungs of the ladders (Metz and Whishaw, 2009). Placing the rungs at uneven distances would provide an even greater challenge. In the case of animals, elevating the horizontal ladder over open space would also increase the consequences of falling and more closely simulate the human experience of walking. In humans, a horizontal ladder task may pose an excessive safety risk, but the effect could be safely approximated by instructing participants to step onto particular targets marked on the floor.

Predictive validity is the demonstration that clinically effective interventions in humans will confer a similar effect in aged rodents with a dual-task deficit (Denayer et al., 2014), or that a novel intervention strategy identified in animals models will offer benefits to older adults. Moving forward, the observation that better aerobic fitness in older adults is associated with a reduced dual-task cost in walking speed during simultaneous treadmill walking and Stroop task performance (Chaparro et al., 2020), indicates that an exercise intervention in rodents could be leveraged to test the predictive validity of a novel rat model of dual-task behavior.

Finally, target validity is the notion that the same underlying mechanisms account for age-related declines in dual-tasking in both rodents and humans. The data presented here, as well as other publications (for review see Udina et al., 2019), implicate age-related alterations in prefrontal cortical activation to dual-tasking impairments. Disruptions in the balance between inhibition and excitation are well documented in the rat prefrontal cortex (Insel et al., 2012; McQuail et al., 2015), and prefrontal cortical activation is elevated when cognitive demand is low and does not appropriately increase to match higher task demand in aged compared to young rats (Hernandez et al., 2018b). Thus, it appears that both aged humans and rats have a reduced dynamic range of prefrontal cortical activity that likely accounts for dual-tasking impairments.

An important modification for future studies will be to make the tasks more difficult to ensure that high functioning older subjects (humans or animals) will be more challenged. The object discrimination task could be made more difficult for humans and animals by assigning objects that are less distinct and therefore harder to discriminate, particularly in aged subjects (Burke et al., 2011; Ryan et al., 2012; Stark et al., 2013; Johnson et al., 2017). By making each task more difficult the overlapping neural circuits contributing to cognitive control of each task will be more fully encumbered, thereby increasing the sensitivity for detecting potential dual-task effects. Another strategy for the additional challenge would be to make the left/right turning decision dependent on a feature of the object discrimination task, rather than using a simple alternation.

## Conclusions

This pilot study provided valuable information that can be used to refine the cross-species dual-task walking protocol, and for identifying behavioral outcome measures that may be sensitive to dual-task cost. This knowledge can be applied to designing future mechanistic and translational studies that seek to identify neurobiological mechanisms affecting dual-task cost with aging.

## Data Availability Statement

The raw data supporting the conclusions of this article will be made available by the authors, without undue reservation.

## Ethics Statement

The studies involving human participants were reviewed and approved by University of Florida Institutional Review Board. The patients/participants provided their written informed consent to participate in this study. The animal study was reviewed and approved by University of Florida Institutional Animal Care and Use Committee.

## Author Contributions

Rodent experiments were designed and conducted by SB and AH, with contributions from QF and SAW. Human experiments were designed by DC, SB and AH, and conducted by DC. All authors contributed to data analysis and interpretation. Manuscript drafting was conducted by DC, SB, and AH, with contributions from SPW, QF, and SAW. All authors contributed to the article and approved the submitted version.

## Conflict of Interest

The authors declare that the research was conducted in the absence of any commercial or financial relationships that could be construed as a potential conflict of interest.
